# *Varroa destructor* resistance to *tau*‐fluvalinate: relationship between *in vitro* phenotypic test and VGSC L925V mutation

**DOI:** 10.1002/ps.7126

**Published:** 2022-09-14

**Authors:** Gabrielle Almecija, Marion Schimmerling, Aurélie Del Cont, Benjamin Poirot, Véronique Duquesne

**Affiliations:** ^1^ Apinov, Scientific Beekeeping and Training Centre Lagord France; ^2^ French Agency for Food, Environmental and Occupational Health and Safety (ANSES), Sophia Antipolis Laboratory, Bee Pathology Unit Sophia Antipolis France

**Keywords:** *Varroa* mite, pyrethroid resistance, bioassay, TaqMan PCR, genotyping

## Abstract

**BACKGROUND:**

*Varroa destructor* is a parasitic mite of the honey bee, *Apis mellifera*. Its presence in colonies can lead to a collapse within a few years. The use of acaricides has become essential to manage the hive infestation. However, the repeated and possibly incorrect use of acaricide treatments, as *tau*‐fluvalinate, has led to the development of resistance. The *in vitro* phenotypic test allows the proportion of susceptible or resistant individuals to be known following an exposure to an active substance. In *Varroa* mites, resistance to *tau*‐fluvalinate is associated with the presence of mutations at the position 925 of the voltage‐gated sodium channel (VGSC).

**RESULTS:**

Here, we compared the results obtained with an *in vitro* phenotypic test against *tau*‐fluvalinate and those obtained with an allelic discrimination assay on 13 treated and untreated *Varroa* populations in France. The correlation between the phenotype and the genetic profile rate is found to be 0.89 *Varroa* mites having resistant phenotypic profile have a probability of 63% to present the L925V mutation (resistance detection reliability). However, 97% of the *Varroa* mites having the susceptible phenotype do not present the L925V mutation (susceptible detection reliability).

**CONCLUSION:**

The L925V mutation explains most of the resistance to *tau*‐fluvalinate in *V. destructor* in the populations tested. However, other mutations or types of resistance may also be involved to explain the survival of *Varroa* mites in the phenotypic test. © 2022 The Authors. *Pest Management Science* published by John Wiley & Sons Ltd on behalf of Society of Chemical Industry.

## Graphical abstract

Phenotypic and molecular susceptibility tests are used to assess the proportion of resistant varroa mites to tau‐fluvalinate. The L925V mutation explained 92% of the resistance to the laboratory‐calculated lethal concentration to kill 90% of the susceptible mite population (LC90). The molecular and phenotypic test could be both used to detect tau‐fluvalinate resistance and advise beekeepers in the choice of the treatment.

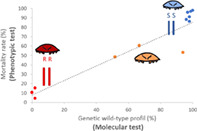

## 1 INTRODUCTION

*Varroa destructor* is a widespread ectoparasitic mite of the honey bee, *Apis mellifera* whose original host is *Apis cerana* ^1^ and is one of the major pests in apiculture. Two haplotypes have infested colonies of *Apis mellifera*. The Korean haplotype found mainly in Europe, and the Japanese haplotype found mainly in the United States and Brazil.^2^, ^3^ *Varroa* mite is responsible for varroosis in bees.^4^ It induces a reduction in the amount of fat bodies in infested bees,^5^ a decrease in worker activity,^6^ immunosuppression and an increase in viral infections.^7^ This leads to a reduction of honey production and an enhanced risk of colony loss.^8^

To manage the infestation by *V. destructor*, various synthetic or organic acaricides are available to beekeepers.^4^, ^9^ Among the different synthetic acaricides, one of the first extensively worldwide used was *tau*‐fluvalinate. *Tau*‐fluvalinate is a member of the pyrethroid class that affects the nervous system of mites by disrupting the voltage‐gated sodium channel (VGSC) activity.^10^ The widespread use of this molecule has led to the development of resistance in *V. destructor* ^11^, ^12^, ^13^ as in many other pests with pyrethroids. Currently, two main mechanisms of resistance have been identified in *V. destructor*: metabolic modifications and molecular substitutions in the channel protein (the major binding site of pyrethroids) and specifically the VGSC protein sequence. Insecticide resistances were initially highlighted by *in vitro* phenotypic tests^11^, ^14^, ^15^, ^16^ and tests assessing the activity of detoxification enzymes.^17^, ^18^

To determine the genetic variations associated with this resistance, the first molecular test was performed using a PCR‐SSCP (polymerase chain reaction‐single strand conformation polymorphism) assay detecting differences in DNA methylation between susceptible and resistant individual mites.^19^ Since then, as observed in other arthropods, *tau*‐fluvalinate resistance has been associated with various mutations located on the VGSC gene, and more specifically on the transmembrane domain II of the protein.^20^ Amino acid substitutions induced by mutations at the VGSC are commonly referred to as ‘knockdown resistance’ (kdr). To date, out of the seven mutation sites previously listed in arthropods,^21^, ^22^ the L925 and M918 sites have been clearly correlated with *V. destuctor*.^20^, ^23^ Various amino acid substitutions can be observed at the L925 locus, with variable frequency and nature according to the countries. For example, in the United States the most frequent mutations are L925M and L925I.^24^ However, the most common mutation observed in Europe is L925V, but the L925I substitution was recently reported in Greece, Turkey and Belgium.^25^, ^26^, ^27^ The mutation L925V was also associated with flumethrin resistance in South America.^28^

Detection of *tau*‐fluvalinate resistance is essential to determine whether a population can be effectively treated with *tau*‐fluvalinate. However, it has been reported that resistance to *tau*‐fluvalinate can decrease over time. The reversion period, the minimum time required to lose the resistance, was estimated to be 4–6 years.^29^ Other more recent studies have demonstrated a loss of resistance in unexposed populations to *tau*‐fluvalinate for several years.^30^, ^31^ Another study showed that no significant variation of L925V mutation frequency was observed when *tau*‐fluvalinate application was increased for many years.^32^ The evolution of resistance to *tau*‐fluvalinate is not yet well understood.

Different methods can be used to monitor mite resistance to *tau*‐fluvalinate. The phenotypic tests allow the detection of a proportion of dead/survivor mites depending on acaricide concentration.^11^, ^14^, ^15^ A specimen surviving a phenotypic test can be considered resistant due to metabolic resistance,^17^, ^33^ target mutation^20^ or physiological phenomenon to decrease exposure (penetration, distribution, excretion).^34^ To explain the resistance observed with the phenotypic tests, different studies have been conducted using molecular tests on specimens from various populations.^23^, ^25^, ^35^ One study demonstrated that mites collected after *tau*‐fluvalinate treatment in the field increased the presence of the L925V mutation.^36^ Studies conducted to define the resistance frequency from molecular testing are often performed on pooled mites^32^, ^37^ and as such, it seems more challenging to establish a relevant correlation. To our knowledge, no study has been performed to demonstrate the relationship between susceptible/resistant mites (phenotypic test) and absence/presence of L925V mutation at individual scale. Here, we conducted a study to compare the phenotypic and genotypic methods to detect resistances to *tau*‐fluvalinate with the aim to know the advantages and limits of each test and proposing these methods to the beekeeping organizations.

## 2 MATERIALS AND METHODS

### 2.1 *Varroa* mite collection

The *Varroa* mites were collected from 13 apiaries in different regions in France. The collection was realized by the ADAs (Association of Beekeeping), veterinarians and beekeepers from 2019 to 2020. Highly infested pieces of brood of 15 cm × 15 cm were removed from one to three colonies in the same apiary. The sample pieces of brood were sent that very day to the laboratory and then placed to the oven at 30 ± 1 °C, RH (relative humidity), 60 ± 10%. The phenotypic tests are performed 1 to 3 days after reception.

### 2.2 History of apiary treatment and selection pressure by Apistan

For each apiary where mites were collected, the treatment history was recorded by the beekeeper. To simplify the analysis of the results, three classes were defined according to the conventional acaricides used (Table 1). *Tau*‐fluvalinate is always used alternately with amitraz to limit resistance (official recommendation in France). The use of a winter treatment (with oxalic acid) was not considered for the classes definition. The treatment class called ‘Amitraz’ was considered only for colonies treated with amitraz in the last 2 years. The ‘Mixte’ treatment class represents colonies treated with both amitraz and *tau*‐fluvalinate (Apistan®) in the last 2 years. The last class corresponds to organic beekeepers which does not use either amitraz nor *tau*‐fluvalinate.

**Table 1:** Population classification according to treatment applications in the previous 2 years

Treatment class	Description	Treatment period
Amitraz	Application of amitraz treatment	2 years consecutively
Mixte	Application of *tau*‐fluvalinate and amitraz treatment	During the two last years
Organic	No application of *tau*‐fluvalinate and amitraz treatment	During minimum 5 years

The class ‘Amitraz’ and ‘Organic’ correspond to mite populations unexposed to *tau*‐fluvalinate while class ‘Mixte’ involves selection pressure with *tau*‐fluvalinate. The proportion of susceptible (SS) and resistant (RR) genetic profile is observed depending the presence of selection pressure by *tau*‐fluvalinate.

### 2.3 Evaluation of mite resistance to *tau*‐fluvalinate *in vitro*

To analyse the resistance to *tau*‐fluvalinate, an *in vitro* phenotypic test was performed. The method was based on the protocol described by Maggi *et al*.^31^, ^38^ In the laboratory, mites were removed from brood. Only adult females were conserved for analysis of *tau*‐fluvalinate resistance. Petri dishes were treated with 1 mL of solution containing *tau*‐fluvalinate (PESTANAL®, Sigma Aldrich, St Louis, MO, USA) at 20 μg/mL (solvent–hexane). This concentration was described as the lethal concentration to kill 90% of the susceptible mite population (LC90) in France.^31^ A control is treated only with hexane. For each population, three to five replicates are carried out for control and for LC90. Therefore, 15 to 20 female mites were placed on the Petri dish. Petri dishes with mites were kept in the oven for 1 h (30 ± 1 °C, RH, 60 ± 10%). *Varroa* mites were then transferred to untreated Petri dishes (with no hexane and no acaricide) with three pupae and placed in oven for 24 h (30 ± 1 °C, RH, 60 ± 10%). Mite mortality was recorded 24 h after the exposure to *tau*‐fluvalinate. After stimulation with tweezers, the mites were divided into two groups: (i) dead mites, including dead and paralysed mites and (ii) surviving mites (mites can move). A corrected mortality (Abbott's correction, 1925) rate was calculated according to Eqn (1) taking into account the observed mortality of the control. The mortality of the mite control (Mortality C) indicated the natural mortality of mites due to handling or transport. If the control mortality was higher than 20%, the test was rejected. Depending on the mean corrected mortality, mite populations are classified into three groups: Susceptible (Mortality ≥ 76%), Moderate Resistance (Mortality 41–75%) and High Resistance (Mortality ≤ 40%).^31^

(1) $$\text{Corrected Mortality}=\frac{\text{Mortality}-\text{Mortality}\;C}{1-\text{Mortality}\;C}$$

After mortality assessment, for each population, dead and surviving *Varroa* mites are separated and divided into Eppendorf tubes and kept dry in the freezer (−20 °C) until the molecular test is performed. Two mites populations were not separate, surviving and dead mites, after the phenotypic test (TFDE and TFDC). In these samples, dead and surviving mites were mixed for the molecular test. Only surviving mites were collected for one population after phenotypic test (AM). For this population, only surviving mites were used in the molecular test.

### 2.4 DNA extraction and genotyping by TaqMan PCR assay

Genomic DNA was extracted from individual adult female mites as described by González‐Cabrera *et al*.^20^ Briefly, mites were placed in a 0.5 μL Eppendorf® tube (one mite per tube), incubated at 99 °C for 3 min in 20 μL of a 0.25 mol L^−1^ of sodium hydroxide (NaOH) solution and were ground using a plastic pestle. Then, 20 μL of a neutralization solution consisting of 0.125 mol L^−1^ hydrochloric acid (HCl), 0.125 mol L^−1^ Tris–HCl, and 0.5% Triton X‐100 was added into each tube. The tubes were incubated as earlier and then centrifuged at 3200 × *g* for 5 min. The supernatant containing the genomic DNA was recovered and stored at −20 °C until used for the assay. The detection of the wild‐type or mutant genetic profile was performed according to the protocol described by Gonzales‐Cabrera *et al*.^20^ for each mite sample. DNA amplification is performed using the forward primer Vd_L925V_F 5’‐CCAAGTCATGGCCAACGTT‐3′ and the reverse primer Vd_L925V_R 5’‐AAGATGATAATTCCCAACACAAAGG‐3′. The two probes Vd_L925V_V (YY‐5’‐TTACCCAGAGCTCC‐3’‐MGB‐EDQ) and Vd_L925V_M (6FAM‐5’‐TTACCCACAGCTCCT‐3’‐MGB‐EDQ) detect the wild‐type and mutant allele, respectively. Briefly, the TaqMan real‐time PCR was performed in a total reaction volume of 20 μL containing 2 μL of genomic DNA, 10 μL of 2× Sso Advanced Universal Probes Supermix (Bio‐Rad, Hercules, CA, USA), 0.32 μM of each primer and 0.2 μM of each probe. The assay was run on a CFX96 Real time PCR (Bio‐Rad). The temperature cycling conditions were as follows: 10 min at 95 °C followed by 40 cycles of 95 °C for 15 s and 60 °C for 45 s. Two control plasmids were obtained by synthetic cloning (GeneCust, Boynes, France). Two fragments of 120 bp with or without the L925V target mutation were synthesized, and cloned in the pUC57 vector at the SmaI cloning site. The recombinant plasmids were verified by restriction enzyme digestion analysis and sequencing. A positive amplification control corresponding to the heterozygote genotype consisting of 5 × 10^4^ copies of plasmid containing the wild‐type target sequence and 5 × 10^4^ copies of plasmid containing the mutated target sequence and a negative control were added in each assay. The allelic profile was determined using the Maestro® analysis software (Bio‐Rad). Briefly, the allelic discrimination is realized by the analysis of the data in relative fluorescence units (RFU) at the selected cycle [cycle threshold (Ct) = 40]. The YY dye was detected for the wild‐type allele while FAM dye was detected for the mutant allele. The data were validated using the results obtained with the negative and positive controls.

### 2.5 VGSC fragment sequencing

Genomic DNA extracted from individual mites, as described earlier, was used to study and verify the mutation site. A partial sequence of the VGSC gene domain II was amplified according to the method described by González‐Cabrera *et al*.^24^ Briefly, a nested PCR was performed on the previously obtained DNA extracts to amplify a final fragment of 484 bp. For the first amplification (PCR1), the primers were 1457iF (5’‐GCTACGTCGCTGTATCTCCC‐3′) and 1973iR (5’‐GCTGTTGTTACCGTGGAGCA‐3′) and for the second amplification (PCR2), the primers were 1479iF (5’‐ACTCTTTCTCCCTCCCTCCC‐3′) and 1963iR (5’‐CCGTGGAGCAAGTTGACC‐3′). The PCR products obtained are checked by migration on an Agilent system. An amount of 10 to 50 ng of PCR product was used to perform the Sanger sequencing reaction using the SeqStudio® instrument (Thermo Fisher Scientific, Waltham, MA, USA). Primers 1479iF and 1963iR were used to run the sequencing PCR. The obtained sequences were aligned and compared to the sequences already available in the databases.

### 2.6 Statistical analysis

Spearman’ correlation index and Pearson's correlation index were used to evaluate the relationship between phenotypic and molecular test. As the *kdr‐*type resistance is described as a recessive trait,^10^ the heterozygous mites (SR) for L925V mutation were considered as susceptible to *tau*‐fluvalinate treatment. The specificity and the sensitivity of the molecular test were defined as Susceptible Detection Reliability and Resistance Detection Reliability, respectively. This two parameters were evaluated compared to the *in vitro* phenotypic test as a ‘gold standard’. The Susceptible Detection Reliability describes the capacity to detect the absence of L925V mutation (defined also as wild‐type genetic profile) in susceptible mite population (dead mites at the LC90). The Susceptible Detection Reliability index is the ratio of the number of susceptible individuals without the mutation to the total number of susceptible individuals (Eqn (2)).

(2) $$\begin{array}{l} \text{Susceptible Detection Reliability}\\ \;=\frac{\text{Number of susceptible individuals without mutation}}{\text{Total number of susceptible individuals}} \end{array}$$

On the contrary, the Resistance Detection Reliability states the capacity of the test to detect the L925V mutation (defined also as mutant genetic profile) for resistant *Varroa* mite population (mites surviving at the LC90). The Resistance Detection Reliability is described by the ratio of number of individuals surviving the LC90 with the mutation L925V to the number of total resistant individuals (Eqn (3)).

(3) $$\begin{array}{l} \text{Resistant Detection Reliability}\\ \;=\frac{\text{Number of resistant individuals with the}\;L925V\;\text{mutation}}{\text{Total number of resistant individuals}} \end{array}$$

The use of predictive values confirms the probability to obtain the correct answer with the molecular test depending on the phenotypic test. The True Resistance Detection (TRD) indicates the probability that *Varroa* mite is truly resistant if the L925V mutation is present (Eqn (4)). The TRD depends on the true‐resistant and the false‐resistant. The true‐resistant are the resistant mites with the L925V mutation. The false‐resistant are the susceptible mites while they have the L925V mutation.

(4) $$\text{True Resistance Detection}\;\left(\mathrm{TRD}\right)=\frac{\text{True resistant}}{\text{True resistant}+\text{False resistant}}$$

The True Susceptible Detection (TSD) indicates the probability that the *Varroa* mite is susceptible to tau‐fluvalinate when L925V mutation is absent. The TSD depends on the true‐susceptible and the false‐susceptible. The true‐susceptible corresponds to susceptible mites which do not present the L925V mutation. The false‐susceptible do not have L925V mutation but are resistant at the LC90 to tau‐fluvalinate.

(5) $$\begin{array}{l} \text{True Susceptible Detection}\left(\mathrm{TSD}\right)\;=\frac{\text{True susceptible}}{\text{True susceptible}+\text{False susceptible}} \end{array}$$

## 3 RESULTS

A total of 13 *Varroa* mite populations were tested with both phenotypic and molecular tests to validate susceptibility or resistance to *tau*‐fluvalinate. Of these 13 populations, 679 mites were tested individually with the molecular test. Table 2 presents the different results obtained with the phenotypic and molecular tests.

**Table 2:** Origin of *Varroa* mites, mite treatment history, phenotype observed at the lethal concentration to kill 90% of the susceptible mite population (LC90) and mortality rate, number of mite for each genotype profile at the position 925 of the VGSC protein and susceptibility rate (SS + SR/total, %)

Apiary	Treatment class	Phenotype^a^	*Tau*‐fluvalinate mortality rate (%)	Genotype^b^				
			*in vitro*	SS	SR	RR	Total	*Tau*‐fluvalinate susceptibility rate (%)
1^c^ (TFDE)	Organic	S	98	58	0	0	58	100
2 (D020819)	Organic	S	97	102	0	1	103	99
3 (VE)	Amitraz	S	96	29	0	1	30	97
4 (VC)	Amitraz	S	91	60	1	0	61	100
5 (JC11)	Organic	S	88	51	0	2	53	96
6 (SP3)	Amitraz	S	86	58	0	0	58	100
7 (17)	Mixte	MR	60	38	6	13	57	77
8^d^ (Am)	Organic	MR	53	15	1	0	16	100
9 (MM2)	Mixte	MR	50	42	1	2	45	95
10 (BV)	Mixte	MR	48	31	4	25	60	58
11^c^ (TFDC)	Organic	HR	15	1	0	45	46	2
12 (PG)	Mixte	HR	10	0	0	36	36	0
13 (LA)	Mixte	HR	4	1	1	54	56	2
Total genotyping rate (%)				71.6	2.1	26.4	100	

^a^ S corresponds to a susceptible *Varroa* mite population, mortality > 76%; MR corresponds to a *Varroa* mite population with moderate resistance, mortality between 41–75%; HR corresponds to a *Varroa* mite population considered highly resistant, mortality ≤ 40%.

^b^ Number of *Varroa* mites of each genotype detected in each population: SS (Homozygous Susceptible), SR (Heterozygous Susceptible‐Resistant) and RR (Homozygous Resistant).

^c^ *Varroa* population not separated after the phenotypic test (susceptible and resistant mites).

^d^ Only the population of living *Varroa* mites, therefore resistant, were tested with the molecular test.

The phenotypic test divided the 13 populations studied into three sensitivity classes described in Almecija *et al*.^31^: six were susceptible (S), four moderately resistant (MR) and three highly resistant (HR). With the phenotypic test, almost half of the tested population (46%) were susceptible (mortality > 75%) to *tau*‐fluvalinate. Out of these samples, the homozygous genetic profile (SS or RR) was more present than heterozygous profile (SR): SS, with 71.4% of the tested mite and RR, with 26.1% of the tested mites). *Varroa* mites with heterozygous profile represented only 2.5% of the samples.

The presence of the L925V mutation seems to explain the *Varroa* mite survival with the phenotypic test. Nevertheless, two *Varroa* mite populations (Am and MM2) considered as moderately resistant with phenotypic test (mortality at the LC90 is between 41% and 75%) have presented a high susceptible genetic profile (SS). In these populations, surviving mites with phenotypic test did not present the L925V mutation.

### 3.1 Phenotyping and genotyping data

**Figure 1:**
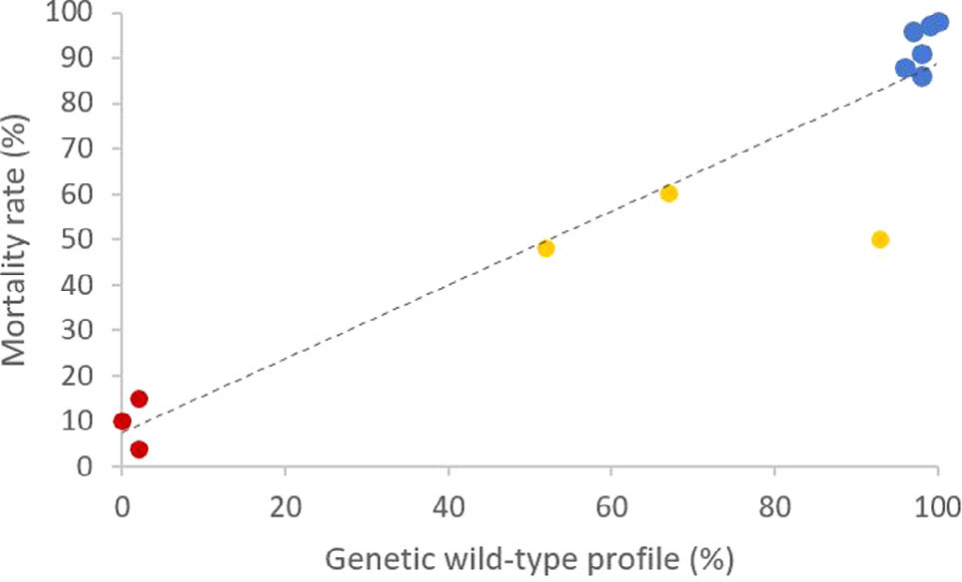
Relation between mortality percentage at the LC90 (phenotypic test) and the genetic wild‐type profile (SS) for each population (*n* = 12). Spearman correlation indicates *R* ^2^ = 0.89. Each point corresponds to a proportion of susceptible mites (phenotypic test) and proportion of wild‐type genetic profile (genotypic test).

Correlation analyses were performed to investigate the relationships between the proportion of resistant *Varroa* mites determined by the phenotypic test and the frequency of detection of the resistant allele by the TaqMan PCR assay. Figure 1 describes the relationship between mortality rate at the LC90 and susceptible (or wild‐type) genetic profile on the 13 populations. The populations detected as susceptible with the phenotypic test present a highly susceptible genetic profile (SS) (Spearman correlation, df = 10, *R* ^2^ = 0.89, *P* = 1.2e‐04) (Fig. 1). If the mite population is detected as resistant with phenotypic test (mortality at the LC90 inferior to 40%), the *Varroa* mite proportion with susceptible genetic profile (no L925V mutation) is very low (< 2%) (Fig. 1).

**Figure 2:**
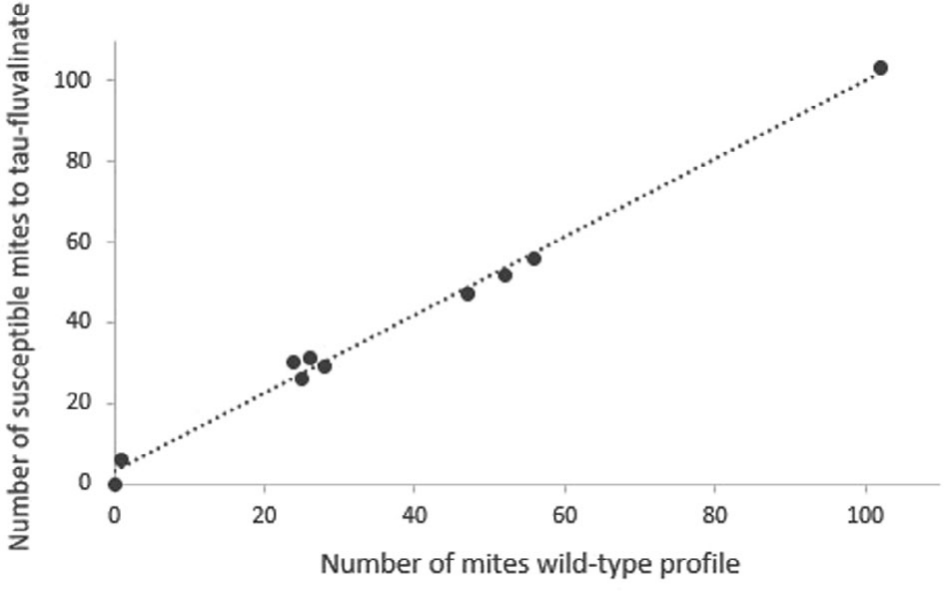
Relation between the number of susceptible mites to fluvalinate at the LC90 and the number of mites with the wild‐type genetic profile (*n* = 10). Pearson correlation, *R* ^2^ = 0.997. Each point corresponds to the number of susceptible mites with the wild‐type genetic profile by population. The population TFDC and TFDE are excluded from the graph because the surviving/dead mites were not separate after the phenotypic test. AM was also excluded because only surviving mites were collected after phenotypic test.

**Figure 3:**
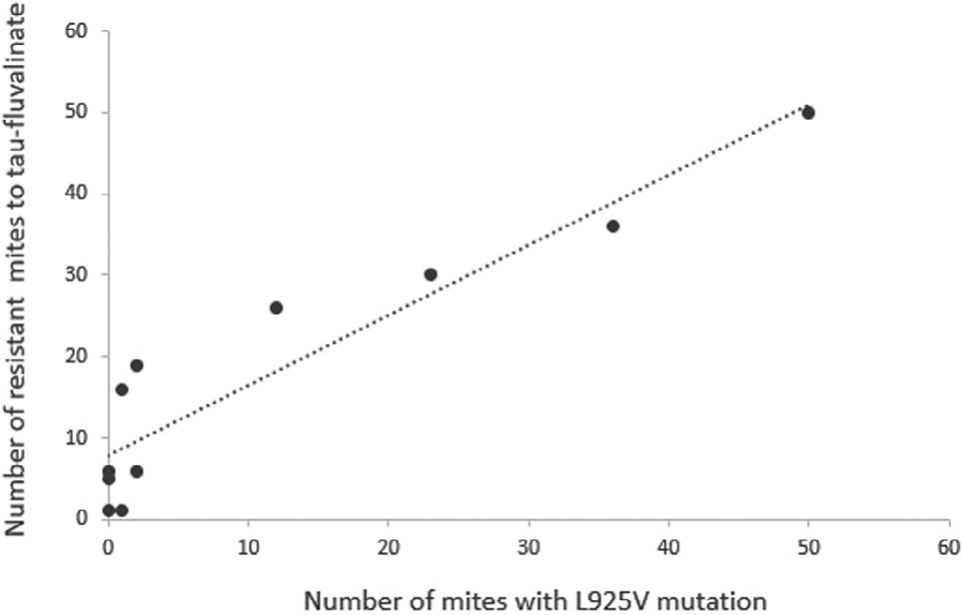
Relation between the number of resistant mites to fluvalinate at the LC90 and the number of mites with the L925V mutation (*n* = 11). Pearson correlation, *R* ^2^ = 0.928. Each point corresponds to the number of resistant mites with the L925V mutation by population. The population TFDE and TFDC were excluded because surviving and dead mites were not separate after the phenotypic test.

The individual comparison between the phenotypic results (Susceptible/Resistant) and the wild type genetic profile (L925V mutation, presence or absence) are shown in Figs 2 and 3. Figure 2 presents the relationship between the number of susceptible mites (phenotypic test) and the number of mites with a wild‐type genetic profile. A linear relation was observed with *R* ^2^ of 0.997. This relationship explained that susceptible mites present the wild‐type genetic profile (Fig. 2) (Pearson correlation, df = 8, *R* ^2^ = 0.997, *P* = 3.33e‐10). On the contrary, resistant mites (surviving mites) did not present the wild‐type profile.

Figure 3 presents the relationship between the number of resistant mites and the number of mites having the L925V mutation. This relation represents a *R* ^2^ of 92.8% (Pearson correlation, df = 9, *R* ^2^ = 0.928, *P* = 3.69e‐05).

### 3.2 Characterization of the molecular test via the *in vitro* bioassay test

The Susceptible Detection Reliability and the Resistance Detection Reliability of the molecular test were calculated to be 96,8% and 62,6%, respectively. The surviving mites at the LC90 considered as resistant mites with the phenotypic test do not consistently have the L925V mutation (Table 3 and Fig. 3).

**Table 3:** Susceptible Detection Reliability and Resistant Detection Reliability of the molecular test for the evaluation of resistance to *tau*‐fluvalinate. The TFDE and TFDC populations were excluded from the analysis as the dead and surviving mites were not separate after the phenotypic test

	Number of *Varroa* with the susceptible phenotype	Number of *Varroa* with the resistant phenotype	Total
Number of *Varroa* with the susceptible genotype (SS and SR)	368	73	441
Number of *Varroa* with the resistant genotype (RR)	12	122	134
Total	380	195	575
Susceptible Detection Reliability^a^	0.97	—	
Resistant detection Reliability^b^	—	0.63	
Confidence interval (95%)	0.95–0.98	0.56–0.69	

^a^ The Susceptible Detection Reliability of the molecular test is defined by the ability to detect susceptible genotyping population among the *Varroa* population susceptible to *tau*‐fluvalinate *in vitro* exposure.

^b^ The Resistant Detection Reliability of the molecular test is defined by the ability to detect resistant genotyping population among the *Varroa* population resistant to *tau*‐fluvalinate *in vitro* exposure.

When *Varroa* mites present the L925V mutation, the probability to be detected resistant with the phenotypic test is 91% (True Resistant Detection). However, when *Varroa* mites do not have the L925V mutation, the probability to be detected susceptible with the phenotypic test is 84% (True Susceptible Detection). The false result rate is therefore 9% and 16% for the detection of mutant and wild‐type profiles, respectively.

### 3.3 Sequencing results

Some results showed discrepancies between the phenotypic and genotypic profile. The genotyping profiles were obtained by targeting only the L925V mutation. This region was sequenced for 46 specimens from the three different populations: JC11 (*n* = 4), D020819 (*n* = 2), LA (*n* = 12), BV (*n* = 12), Am (*n* = 8) and MM2 (*n* = 8). Four phenotype/genotype combinations were observed: Susceptible/Wild‐type, Susceptible/Mutant, Resistant/Wild‐type and Resistant/Mutant (Table 4). All the specimens (*n* = 27) with wild‐type genetic profile did not show mutations at 918 and 925 loci. However, among these specimens, 25 were resistant when exposed to *tau*‐fluvalinate using the *in vitro* test. However, mutant genotype of 19 specimens tested present the substitution L925V at the L925 locus. In addition, all of them also have a substitution at the 918 locus, which is not targeted by the molecular assay used in this study.

**Table 4:** Sequencing results of the voltage‐gated sodium channel (VGSC) mutation site from susceptible and resistant mites to *tau*‐fluvalinate in relation with the genotypic profile

*In vitro* phenotype	L925 genotype profile	Number of mites sequenced	kdr‐Type alleles	DDBJ accession numbers
S^a^	Wild‐type	2	918 M/M (wt) + 925 L/L (wt)	LC685877/LC685878
S	Mutant	8	918 L/L + 925 V/V	LC685879 to LC685886
R^b^	Wild‐type	25	918 M/M (wt) + 925 L/L (wt)	LC685887 to LC685911
R	Mutant	11	918 L/L + 925 V/V	LC685912 to LC685922

^a^ Susceptible: dead mites after exposition with *tau*‐fluvalinate at the lethal concentration to kill 90% of the susceptible mite population (LC90).

^b^ Resistant: surviving mites after exposition to *tau*‐fluvalinate at the LC90.

### 3.4 Selection pressure following Apistan treatment

**Figure 4:**
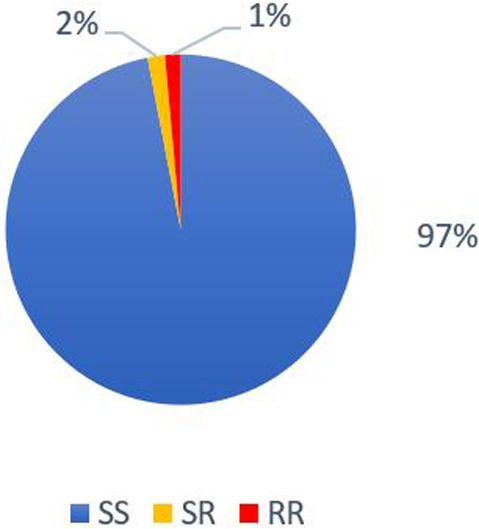
Distribution of the genotypes for L925V mutation for colonies not treated with Apistan® in the last 2 years (Organic and Amitraz classes). The SS corresponds to homozygous susceptible profile. The RR correspond to the homozygous resistant profile and the RS are heterozygous mites.

**Figure 5:**
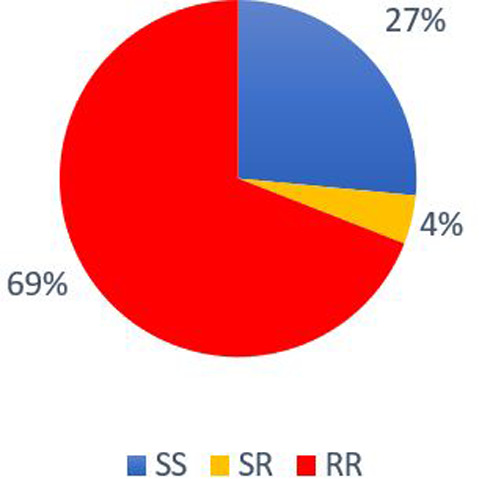
Distribution of the genotypes for L925V mutation for colonies treated with Apistan® in the last 2 years (Mixte class). The SS corresponds to homozygous susceptible profile. The RR correspond to the homozygous resistant profile and the RS are heterozygous mites.

The active substance of Apistan® treatment is *tau*‐fluvalinate. In this study, we established the impact of Apistan® treatment on the L925V mutation development. When *Varroa* mite populations had no contact with Apistan® for a minimum 2 years (Amitraz and Organic classes), the mites presented a mean wild‐type genetic profile of 97 ± 0.86% (*n* = 8). For these populations, the mean mortality rate at the LC90 was 82.75 ± 6.91% (*n* = 8) (Fig. 4). In contrast, the colonies treated with Apistan® in the last 2 years (Mixte class), had a lower wild‐type genetic profile of 27 ± 15.46% (*n* = 5) (Fig. 5). For these populations, the mean mortality rate at the LC90 was 27.4 ± 11.16% (*n* = 5). On the sampled populations, the use of Apistan® was closely associated with the occurrence of the L925V mutation.

## 4 DISCUSSION

In the present study, two different methods used to evaluate the resistance phenomena of *Varroa* mites were compared on populations with different treatment history. The use of phenotypic tests can highlight the presence of *Varroa* mite resistance to a concentration of *tau*‐fluvalinate, which is lethal for susceptible individuals. The molecular test provides additional information on the nature of the resistance, here the L925V mutation. This study demonstrated a strong correlation between the two methods used to assess the level of susceptibility or resistance of parasites to *tau*‐fluvalinate. The results obtained made it possible to highlight the linear relationship between the mortality obtained with the phenotypic test and the susceptible profile of *Varroa* mites with the molecular test (Figs 1 and 2). This relation was also observed with pooled mite sampled and with another phenotypic test.^32^ The choice of the LC90 for phenotypic test seems to improve the relation between phenotypic and molecular test (Fig. 1). Indeed, the percentage of mortality obtained at the LC90 is strongly correlated with the percentage of the susceptible genetic profile of the population (Fig. 1). Susceptible individuals therefore do not present the L925V mutation (Fig. 2). The molecular test has confirmed the observations made from the phenotypic test.

In particular, the L925V mutation can explain the resistant phenotype for 63% of the specimens tested (Table 3). Other type of resistance can explain the survival of the mites in phenotypic test as metabolic resistance or other mutations. Metabolic resistance involving three enzymes in insecticide detoxification^39^, ^40^, ^41^ or cuticular resistance^42^, ^43^, ^44^ have been described and studied in other arthropods. However, other substitutions at position 925 are involved in the *tau*‐fluvalinate mutation in *Varroa* such as the majority of L925M and L925I in the United States,^24^ and the majority of the L925I mutation in Greece.^25^ Here, the sequencing carried out on *Varroa* mites did not reveal any new substitution already described at position 925. However, in addition to this substitution at site 925, the M918 substitution was observed for the first time in France as already described in some specimens in Spain.^45^ The emergence of this new mutation occurred in Spain as early as 2018. This same mutation at the 918 locus was not observed in the few French samples collected in 2014 and 2017 and tested in the study cited later.^23^ As observed in Spain, this mutation seems to appear between 2018 and 2019 in France increasing the character of resistance of mites. The M918L mutation was found in all sequenced mite DNAs that had the L925V mutant profile. In other words, the M918L mutation alone was not observed in our study, even in mites bearing combined phenotype resistant/genotype wild‐type profiles. These two substitutions would thus seem to be the result of a strong selection pressure occurring in French apiaries.

However, our results showed that the absence of L925V mutation does not indicate an absence of *Varroa* mite resistance to *tau*‐fluvalinate (Fig. 3). Indeed, 35% of mites surviving to *in vitro tau*‐fluvalinate exposure did not bear either the M918L or L925V mutation. This is in support of the presence of different resistance types within the same *Varroa* population. Furthermore, the use of one mutation as L925V could also underestimate resistance to *tau*‐fluvalinate. A preliminary study carried out in Italy^30^ proposed a biochemical test targeting esterase activity in order to supplement molecular resistance data and increase the capacity to detect resistance. However, the authors have not yet demonstrated the relationship between the increase in this detoxification activity of acaricides and the resistance of *Varroa* mites to *tau*‐fluvalinate, in particular. In fact, metabolic resistance is not well understood with *Varroa* mites. The activities of different enzymes were evaluated as esterase activity, monooxygenase, cytochrome P450 to explain fluvalinate resistance. While some studies imply the oxygenase, acetylcholinesterase or cytochrome P450^18^, ^46^ other did not find any correlation with resistance to fluvalinate with *Varroa*.^17^, ^33^

To obtain the most representative proportion of the *Varroa* mite population in the colony, the sample size to be tested is an important parameter and must be large enough. The survival of *Varroa* mites at the LC90 or the presence of a mutation at the L925V indicates a risk of treatment failure. A study showed that the susceptibility of *Varroa* mites to amitraz with a phenotypic test was homogeneous within a hive but could in particular cases show significant differences from one hive to another.^31^ The phenomena of re‐infestations due to looting could explain these observations.^4^ The level of sampling (hive, apiary) and the sample size remains to be defined for the molecular test to be carried out routinely. In this context, the TFDC sample, from a beekeeper using only oxalic acid, was brought to our attention. Indeed, *Varroa* mites presented high resistance to *tau*‐fluvalinate (2% susceptible genetic profile). This observation can be explained by a re‐infestation of a colony highly resistant to *tau*‐fluvalinate,^48^ by drift phenomena during heavy honey flows^47^ or by contamination of the environment of the colony by pyrethroids and especially from waxes.^35^ The test should be performed before the beekeeper considers and orders the treatment. However, the infestation may be too low in the months before treatment to realize a phenotypic test. In this case, molecular test could allow to analyse mite susceptibility in spring. The phenotypic test can be applied the year before or just a few weeks before treatment.

The number of heterozygous individuals observed in the present study is very low (less than 3%) and comparable to those observed in the literature.^25^, ^28^, ^30^, ^48^ The haplodiploid mode of reproduction of *V. destructor* may explain this low frequency of heterozygosity.^25^, ^49^ Heterozygous specimens are considered susceptible for resistance because the ‘kdr’ mutation is known to be a recessive character.^24^

On our samples, 69% of *Varroa* mites exhibited the homozygous mutant genotype following recent treatment with *tau*‐fluvalinate (< 2 years). The L925V mutation therefore seems to be the most frequent to explain resistance to *tau*‐fluvalinate, as already shown by González‐Cabrera *et al*. in 2018^36^ for Europe. In the absence of *tau‐*fluvalinate treatment (> 2 years), the susceptible genetic profile is present at 97% (Fig. 4). This seems to imply that *Varroa* mites can regain their sensitivity to *tau*‐fluvalinate quite quickly (after 2 years minimum without *tau*‐fluvalinate treatment for our study). This result is more encouraging than that presented for populations originating from the United States where 45% of populations without contact with *tau*‐fluvalinate presented the homozygous resistant genotype.^24^ Other studies have also highlighted the absence of mutant *Varroa* mites in several populations since the discontinuation of the use of Apistan®.^29^, ^30^ This period during which *Varroa* mites recover their sensitivity is called the reversion period. Today, alternate control strategies are based on current knowledge of the reversion period for *tau*‐fluvalinate. One study had notably defined a period of 4 years to go from a resistant to susceptible population.^29^

## 5 CONCLUSION

While the phenotypic test provides information on the general state of susceptibility of an individual or a population, the molecular test makes it possible to determine the nature of the resistance. In this experiment, the L925V mutation mainly explains the presence of resistant *Varroa* mite in the sampled populations. Nevertheless, it is confirmed that other resistance mechanisms can also lead to *Varroa* mite resistance to *tau*‐fluvalinate. The molecular test has the advantage to be performed on dead *Varroa* mites and to be fast. The collection of samples is therefore easier compared to the phenotypic test which is time‐consuming as it requires live *Varroa* mites. In addition, the cost of the molecular test would be lower than the phenotypic test, as long as the test could be performed on pooled samples of mites.^27^, ^32^ Further investigations may provide new elements to optimize these tools as the number of hives and the number of mites sampled. To conclude, the availability of phenotypic and molecular tests for the beekeeping sector is of definite interest in the implementation of prophylactic treatments and monitoring of resistance management strategies.

## AUTHOR CONTRIBUTION

Gabrielle Almecija: developed the *in vitro* model, analysed all the data, evaluated mite resistance in the laboratory and contributed to the drafting of the manuscript, critical revision for important intellectual content. Marion Schimmerling: made substantial contributions to the acquisition, analysis, and interpretation of molecular data. Aurélie Delcont: supervised molecular analysis, contributions to the acquisition, analysis, and interpretation of molecular data. Benjamin Poirot: drafting of the manuscript and critical revision for important intellectual content. Véronique Duquesne: supervised and analysed the molecular data, drafting of the manuscript and critical revision for important intellectual content.

## CONFLICT OF INTEREST

The authors declare that there is no conflict of interests in this study.

## Data Availability

The data that support the findings of this study are available from the corresponding author upon reasonable request." cd_value_code="text
